# Zeolite Catalysts Prepared with Maximum Brønsted Acidity Reveal a Dominant Contribution from Inaccessible Sites

**DOI:** 10.1021/jacs.6c10814

**Published:** 2026-06-26

**Authors:** Omio Rani Das, Ismaeel Alalq, Jacob Crouch, Anya Zornes, Steven Crossley, Bin Wang, Jeffery L. White

**Affiliations:** † School of Chemical Engineering, 7618 Oklahoma State University, Stillwater, Oklahoma 74078, United States; ‡ School of Sustainable Chemical, Materials, and Biological Engineering, 6187 University of Oklahoma, Norman, Oklahoma 73019, United States

## Abstract

Zeolite Y catalysts contain Brønsted acid sites in accessible large pores, known as supercages, and in sterically inaccessible small pores within sodalite cages. Access to acid sites within sodalite units is precluded due to their 0.26 nm cage windows, which are smaller than the critical diameters of relevant hydrocarbons. By preparing a series of HY catalysts with different acid site densities, including for the first time reported those with theoretical maximum acid site density, the number of acid sites in the inaccessible sodalite cages is shown to be a dominant factor in reactivity for catalysts in which the structural integrity of the sodalite unit is preserved. Using a combination of catalyst preparation, spectroscopy, isotopic exchange measurements with bulky hydrocarbons, and high-temperature isooctane cracking experiments on catalysts with fixed supercage proton amounts but varying sodalite proton concentrations, the dominant contribution of sodalite cage acid sites is quantified. DFT calculations suggest that a plausible mechanism involves framework flexibility in the form of site exchange via rotation of sodalite protons into supercages. In addition to revealing that commonly used acid site density measurements and turnover frequency calculations may exclude contributions from important sites, these data demonstrate that BASs in intact sodalite cages can define and control catalyst reactivity in HY catalysts. Large increases in catalyst activity are obtained by exchanging La^3+^ cations into sodalite cages with maximum acid site densities. These findings extend beyond HY catalysts, as they imply that other zeolite catalysts with sterically occluded acid sites may be tuned for improved performance.

## Graphical abstract

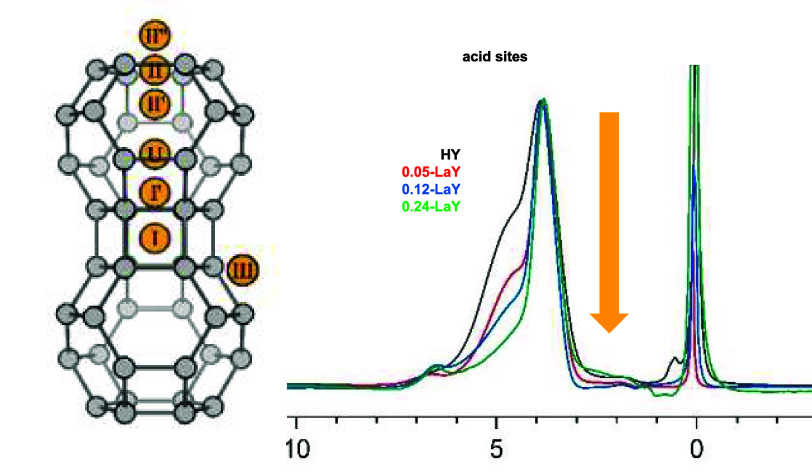

## Introduction

The faujasite class of zeolites includes zeolite Y, which in its acidic and rare-earth exchanged forms is the most widely used industrial zeolite catalyst family for hydrocarbon conversion via fluid catalytic cracking processes.^1–3^ As eloquently described by Vogt and Weckhuysen in a recent comprehensive review, this “grand old lady” of zeolite catalysis continues to impact current and future applications in fuels and materials.^1^ Zeolite Y, a large-pore zeolite, has a three-dimensional microporous framework characterized by 12-membered ring supercages and 6-membered ring sodalite cages, yielding Brønsted acid site (BAS) locations based on framework Al incorporation situated in 1.24 and 0.66 nm diameter cavities, respectively.^4,5^ While the sodalite cages are themselves large enough to accommodate some hydrocarbons, the 0.26 nm 6-membered ring openings into the cages prevent ingress. To increase access to these sterically inaccessible BASs, elegant methods have been reported for opening up the six-membered sodalite cage rings in a controlled fashion to improve hydrocarbon reactions.^6–8^ Controlled procedures for sodalite cage opening are attractive, since the faujasite framework in its acidic form is known to be susceptible to hydrolysis by water, including ambient moisture.^9,10^ Recently, it has been shown through quantitative spectroscopy experiments that hydrolysis preferentially occurs at sodalite BASs in HY,^11^ and importantly, several reports have demonstrated that some structures formed via framework hydrolysis at BASs can be reversed through controlled ammonia treatments.^12–14^ With respect to the latter, seminal papers by van Bokhoven and co-workers have clearly shown that species that are often erroneously assigned as six-coordinate extra-framework Al species (EFAl) in many literature studies are actually framework bound, and cannot give rise to Lewis acidity in the absence of hydrothermal treatments.^12–14^ Framework hydrolysis at elevated temperatures leads to dealumination and formation of EFAl whose exact structures depend not only on experimental conditions but overall thermal histories, and which may also contain sites that exhibit Lewis acid character yielding an overall catalyst with a complex acid function that can be difficult to characterize.^15–17^

An HY zeolite catalyst whose framework BAS density was equal to the theoretical maximum allowed by framework Al content could, when compared to other defect-free HYs with lower BAS density, reveal whether acid sites in physically inaccessible intact sodalite cages were relevant to overall catalyst performance. The typical as-synthesized Y zeolite has a Si/Al near 2.6, with small variations. To date, no reports of a total framework BAS density ([BAS]) approaching the ca. 4–4.3 mmol/g expected for these Si/Al have appeared in the literature. The majority of reported [BAS] for HY catalysts with Si/Al near 2.6 range from ca. 0.5 to 2 mmol/g,^6,14,15,18–22^ with some reports slightly above 3 mmol/g based on infrared spectroscopy.^7^ In many published cases, structural heterogeneity, the possibility of EFAl species with either synergistic or deleterious impacts, and potential Lewis acid function, all of which can depend on overall catalyst thermal and moisture history, complicates data interpretation and precludes accurate catalyst structure and reactivity assessments.^23^ Thus, a logical basis for assessing sodalite BAS contributions requires starting with controlled catalysts whose sodalite and supercage BAS densities can be easily modified via nondestructive postsynthetic treatments, and measuring their impacts on hydrocarbon reactivity.

The preparation, characterization, and reactivity of several defect-free HY catalysts with varying BAS density up to the maximum value near 4.3 mmol/g expected based on Al content in the catalyst is reported here. In a systematic approach, the [BAS] in supercages is held constant as the sodalite [BAS] varies. Surprisingly, in both room-temperature isotopic exchange experiments and high-temperature cracking experiments involving bulky hydrocarbon reagents in the absence of moisture or polar compounds, it is the concentration of Brønsted acid sites in the intact sodalite cage ([H^+^_SD_]) that dictates conversion and turnover frequencies. The latter increase several times with increasing [H^+^_SD_] *for a fixed concentration of Brønsted acid sites in the supercages* ([H^+^_SC_]). Such a result is in no way expected based on the conventional view of molecular adsorption at an accessible acid site. As a result of this surprising impact of confined acid sites in sodalite cages, controlled replacement of [H^+^_SD_] with the rare earth cation La^3+^ was used to further elucidate the impact of sodalite sites. Again, while holding the concentration [H^+^_SC_] constant and in the absence of framework degradation, controlled incorporation of La^3+^ exclusively in sodalite units results in an additional several-fold increase in conversion and reaction rate constants above their maximum values measured for the purely acidic catalyst with maximum [H^+^_SD_]. The origin of this unexpectedly dominant contribution from acid sites in sterically inaccessible locations is discussed, as well as implications for other zeolites that also contain inaccessible acid sites.

## Experimental Section

### Catalyst Samples

Zeolite NH_4_Y (CBV300) with a nominal Si/Al = 2.6 was obtained from Zeolyst International. Catalysts denoted as **HY** were prepared from NH_4_Y in a glass reactor by stepwise vacuum dehydration, with a pressure less than 1 × 10^–4^ Torr maintained throughout the process, heating at 0.5 °C/min to 100 °C, holding for 2 h, heating at 2 °C/min to 450 °C, and holding for 12 h. Catalysts denoted as **HY-3x** and **HY-5x** were prepared from the parent NH_4_Y, but subjected to either 3 or 5 additional ion-exchange steps with 1 M NH_4_Cl, respectively. The aqueous ion-exchange solutions were held at 80 °C for 3 h for each exchange step. Following the final exchange, samples were filtered and vacuum-oven-dried overnight at 80 °C, and then placed in the glass reactor and subjected to the same slow-heating vacuum dehydration sequence used to prepare HY. The catalyst denoted as **HY-Na-1x** was prepared from the parent NH_4_Y (CBV300) by following the same aqueous ion-exchange procedure described above, using 0.1 M NaCl instead of NH_4_Cl, for one exchange sequence. All prepared catalysts were stored in vacuum or inert atmosphere prior to subsequent spectroscopy or reactor studies.

**LaHY-5x** was prepared from the NH_4_Y-5x by exchanging 500 mg NH_4_Y-5x in 10 mL aqueous 0.01 M La­(NO_3_)_3_ solutions with concentration corresponding to 0.05 La:Al molar ratio for 4 h at 90 °C. Samples were vacuum filtered and rinsed with DI water, then dried in a vacuum oven at 80 °C overnight. The samples were then fully dehydrated by stepwise vacuum dehydration, with pressure maintained below 1 × 10^–4^ Torr throughout, heating at 0.5 °C/min to 350 °C, holding for 10 min, then heating at 2 °C/min to 550 °C and holding for 2 h.

### NMR Hardware and Sample Packing

All NMR experiments on the solid catalysts were conducted at 9.4 T with a Bruker Avance II console using a 4 mm double-resonance MAS probe. Samples were packed in ZrO_2_ rotors, under either ambient air (^27^Al and ^29^Si NMR), or in an inert argon gas atmosphere using a VAC atmosphere glovebox (^1^H NMR).

### ^1^H NMR Experiments

^1^H NMR spectra were acquired on fully dehydrated catalysts using a single 90° excitation pulse of 4 μs. In all cases, 64 transients were obtained with a recycle delay of 60 s, which is in excess of 5 times the longest relaxation time observed, with a spinning speed of 10 kHz. Pulse durations and chemical shifts were calibrated using hexamethylbenzene. For quantitative spin-counting experiments, measured amounts of vacuum-dehydrated sample and an inert quantitation standard poly­(dimethylsiloxane) (PDMS) were confined to the middle third of a 4 mm ZrO_2_ rotor as previously described in detail.^24^

### ^27^Al NMR Experiments

^27^Al NMR spectra were acquired on ambient-air hydrated samples with a 15° pulse of 0.6 μs and a recycle delay of 0.2 s for 4096 transients at a spinning speed of 10 kHz. Pulse durations and chemical shifts were calibrated using a 0.1 M aqueous solution of Al­(NO_3_)_3_.

### ^29^Si NMR Experiments

^29^Si NMR spectra were acquired on ambient-air hydrated samples with a 90° pulse of 4.8 μs. All spectra were acquired for 1024 transients with a recycle delay of 60 s and a spinning speed of 10 kHz. Pulse durations and chemical shifts were calibrated using PDMS.

### XRD

Powder X-ray diffraction data were acquired at ambient humidity using a Phillips X-ray Diffractometer (Phillips PW 3710 MPD, PW2233/20 X-ray tube, copper tube detector, wavelength 1.5418 Å) which operated at 45 kW and 40 mA. Diffractograms were obtained with 2θ ranging from 2° to 45°, and with a diffractometer difference of 0.02°.

### Elemental Analysis

Elemental analysis to determine the weight percent (wt %) of Si, Al, La, and Na in the exchanged samples was provided by Galbraith Laboratories using the GLI ME-70 procedure.

### In Situ Fourier Transform Infrared (FTIR) Spectroscopy and High-Temperature Adsorption

Probe molecule sorption experiments were performed using a PerkinElmer Spectrum 100 spectrometer equipped with a high-temperature diffuse reflectance infrared Fourier transform spectroscopy (DRIFTS) Praying Mantis cell from Harrick Scientific. Background spectra were collected using KBr (FT-IR grade, ≥99% trace metals basis, Sigma-Aldrich). Approximately 100 mg of ammonium-form catalyst sample was loaded into the DRIFTS cell and calcined in situ under 30 SCCM He flow by heating to 450 °C at a ramp rate of 5 °C/min and holding for 4 h. The sample was subsequently cooled to 25 °C prior to the introduction of 2,6-di*tert*-butylpyridine (DTBPy) for the in situ adsorption experiment. Multiple 5 μL injections of DTBPy were introduced at 25 °C while spectra were collected at different saturation levels to monitor the interaction of the probe with Brønsted acid active sites located in different environments within the Y zeolite samples. After complete saturation, the temperature was increased to 450 °C and maintained for 1h under He flow to partially desorb DTBPy from the acid sites. The sample was then cooled back to 25 °C and the spectra were collected for comparison with the fully saturated state.

### Kinetic Measurements

The impact of sodalite acid site concentration on catalyst activity was measured using two model reactions: isooctane (2,2′,4-trimethylpentane) cracking in a continuous-flow microreactor at 400 °C and in situ H/D exchange solid-state NMR experiments using toluene-d_8_ as the reagent at room temperature. For isooctane cracking reactions, catalyst samples were pelletized to a size of 250–425 μm with a catalyst mass of 20 mg. For iso-conversion experiments probing selectivity, catalyst mass was varied from 15 to 50 mg as necessary to achieve a fixed ca. 8% conversion. Prior to reaction, catalysts samples were pretreated to remove any moisture present by vacuum heating (25 Torr) to 100 °C at 0.5°/min, holding at 100 °C for 2 h, heating to 400 °C at 2°/min and holding at 400 °C for 12 h. After calcination, the vacuum was gradually removed by turning off the vacuum pump while flowing 20 SCCM helium. The reaction was conducted at 400 °C by feeding 0.5 mL/h i-octane with 80 SCCM helium. Each run was conducted for approximately 6 h, with analysis of reaction products using a Shimadzu GC-2010 Plus GC-MS equipped with HP-PLOT/Al_2_O_3_/S column to determine conversion and product selectivity.

In-situ H/D exchange experiments were carried out at room-temperature using catalyst samples loosely packed in a ZrO_2_ rotor in an inert argon atmosphere, which were then evacuated under vacuum. Toluene-d_8_ was adsorbed onto the catalyst such that for all samples a 1:1 molar ratio of reactant to the total number of Brønsted acid sites was achieved. The rotor was then sealed using a Teflon spacer and kept in a liquid nitrogen bath until the sealed rotor was inserted into the NMR probe. ^1^H NMR spectra were then obtained at room temperature over the course of the reaction, with the integrated area of each peak correlating directly to the concentration of that species. Rate constants for the reaction were determined using a least-squares fit of the growth of the toluene peak area *T*(*t*) during the short-time initial-rate region of the proton/deuterium exchange curve using the equations stated in the text and relevant figure captions.

### DFT Calculations

Density Functional Theory (DFT) calculations were performed using the Vienna Ab-initio Simulation Package (VASP)^25^ with projector augmented wave (PAW) potentials^26^ and the Perdew–Burke–Ernzerhof (PBE) functional^27^ paired with DFT-D3 to approximate dispersion.^28^ KPOINTS were set to 3 × 3 × 3 to sample the Brillouin zone. Geometry calculations were carried out using a kinetic cutoff energy of 500 eV with the structure relaxed until the atomic net force was less than 0.02 eV Å^–1^. A rhombohedral primitive cell was employed for zeolite Y with a SAR equal to three (48 T sites), with the aluminum placed into the 4332 arrangement, each number representing the amount of aluminum in each hexagonal bridge, based on comparison of NMR data to DFT estimations as discussed in a prior study.^29^ Transition states were located using Nudged Elastic Band (NEB) and Dimer methods.^30–32^

## Results and Discussion

Potential synergistic contributions between framework BASs and species within the internal volume of a zeolite catalyst, but not within the covalent framework itself, are currently the subject of extensive discussions within the heterogeneous catalysis literature.^23,33–38^ The most widely discussed framework/nonframework synergies involve BASs interacting with aluminum oxides or aluminum hydroxides created by framework hydrolysis. Extra-framework alumina or aluminols (EFAl) may be formed either in the catalyst preparation steps or after moisture exposure during use. EFAl species should not exist when the number of BASs equals the amount of aluminum measured from elemental analysis, indicating that catalyst preparation steps do not cause framework Al hydrolysis and that each Al is in the framework, creating the necessary charge-balancing proton site. Achieving this can be difficult for high-Al content catalysts like that typically encountered for zeolite Y catalysts, due to their inherent framework instability in the presence of moisture. However, if such a catalyst can be prepared, then key uncertainties may be eliminated in terms of structure–reactivity analyses. Mechanistic understanding and kinetic modeling can be assessed based only on Brønsted acidity; Lewis acid contributions can be ignored. The role of BASs as a function of their location can be determined, and specifically, contributions from those BASs within sodalite units can be interrogated with respect to their steric inaccessibility.

### Acid Site Identification, Quantification, and Structural Integrity

Several different techniques are routinely employed for determining the number of Brønsted acid sites (BASs) in zeolite HY, with a wide range of values reported based on the method used and potential for structural heterogeneity due to different catalyst histories.^18,19,39–42^ Preferred counting methods historically involve probe molecules that only adsorb at supercage acid sites, e.g., isopropyl amine or pyridine, given the inaccessibility of sodalite acid sites within their 0.26 nm 6-membered ring openings. As one of the chief experimental goals of this work is to measure and understand the impact of BASs confined within intact sodalite units on hydrocarbon reactivity, a noninvasive method for BAS counting that does not employ any type of probe molecule while simultaneously providing absolute species quantitation is required. Multiple reports have recently shown that solid-state ^1^H MAS NMR methods employing an inert spin-counting standard provide accurate, reproducible, noninvasive, and site-specific BAS concentrations in dry zeolite catalysts, silicas, and SAPOs.^24,43,44^ It will be demonstrated below that this experimental approach quantitatively resolves and measures BAS types H^+^_SD_ and H^+^_SC_ without ever exposing the catalyst to moisture, thereby ensuring that no framework degradation occurs following synthesis, exchange, and dehydration. Indeed, if Si–O–Al bonds in the sodalite cages undergo hydrolysis, distinct signals arise indicating framework degradation. This distinctive experimental capability is critical to determining what, if any, role the BASs within the sodalite cages play on hydrocarbon conversion, since it quantifies the species comprising the initial Brønsted acidity in the working HY catalyst.

Figure 1 shows representative quantitative ^1^H MAS NMR spectra for different HY zeolite catalysts. The parent catalyst, denoted HY, is used as the source for preparing new catalysts HY-3x and HY-5x as described in the Experimental Section. Stated simply, HY-3x and HY-5x are prepared by completing three (3x) and five (5x), respectively, ammonium chloride exchange steps on the parent NH_4_Y catalyst. The catalyst labeled as HY-Na-1x was prepared via a one-time exchange of the parent NH_4_Y catalyst with NaCl prior to controlled vacuum dehydration. Prior to use or spectroscopic investigation, all catalysts were subjected to the slow-ramp rate vacuum dehydration protocol detailed in the Experimental Section. Figure 1 shows that all three HY catalysts are characterized by two clearly resolved proton populations, denoted as [H^+^_SC_] and [H^+^_SD_] for the acidic protons in the supercages (3.7 ppm) and sodalite cages (4.6 ppm), respectively. The narrow signal near 0.1 ppm is the inert standard poly­(dimethylsiloxane) (PDMS), added as an internal spin-counting standard and previously shown to provide quantitative and noninvasive quantification of protons in zeolites and zeotype materials.^24^ Importantly, no signals are observed in the 1–3 ppm region of the spectrum, clearly indicating that no internal defect silanols (SiOH) or extraframework and/or internal defect aluminols (AlOH) are formed by the multiple ammonium exchange steps, subsequent dehydration, and inert atmosphere data acquisition. The sensitivity of the ^1^H MAS method to these defects is high, as shown by comparing a representative standard spectrum in Figure S1 to that in Figure S2 acquired after moisture exposure, or following the established literature procedure for opening up HY sodalite cages using NH_4_F etching (Figure S3).^6^ In each of these cases clear signals are observed in the 1–3 ppm region, as expected. The fact that the three HY catalysts maintain all structural integrity is confirmed by measured Si/Al of 2.6–2.7 using both elemental analysis (Table 1) and ^29^Si MAS NMR (Figure S4), 97–99% crystallinity via X-ray diffraction (Figure S5), and high surface areas via nitrogen BET analysis. Table 1 summarizes these routine characterization data, that when taken together with the spectra in Figures 1 and S1 and S2 confirm the preparation of defect-free HY catalysts.

**Figure 1:**
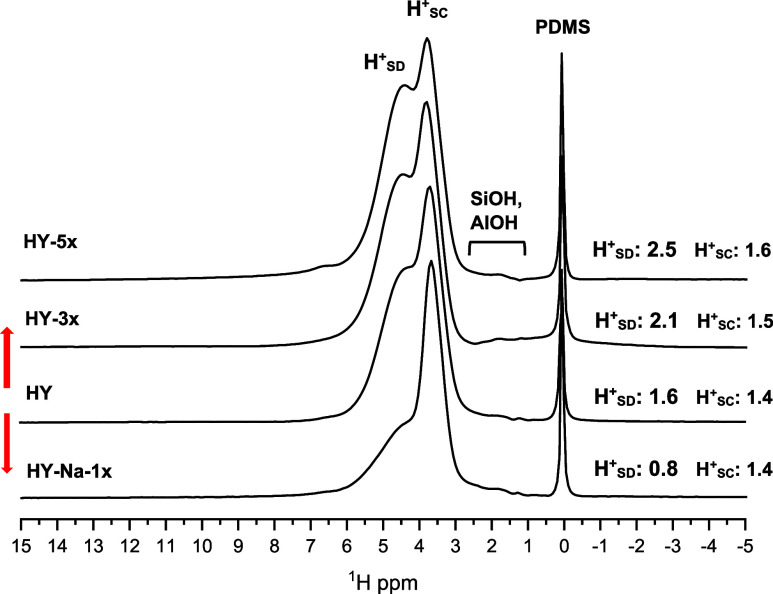
^1^H MAS NMR spectra and accompanying H^+^ concentrations in units of (mmol/g cat) of the several HY catalysts prepared from the parent NH_4_Y via low ramp-rate vacuum dehydration, showing varying [H^+^_SD_] and essentially fixed [H^+^_SC_]. As labeled, **(HY):** parent commercial catalyst; (**HY-3x):** same as HY but following a three-time NH_4_Cl exchange prior to controlled vacuum dehydration; **(HY-5x):** same as parent catalyst but following a five-time NH_4_Cl exchange prior to controlled vacuum. **(HY-Na-1x):** same as parent catalyst but following a one-time exchange with NaCl prior to controlled vacuum dehydration. Measured [H^+^_SC_] and [H^+^_SD_] concentrations in (mmol H^+^/g catalyst) are reported on the right for each catalyst. PDMS denotes the internal spin-counting standard polydimethylsiloxane. Note the absence of SiOH and/or AlOH intensity in the 1–3 ppm region. Associated ^27^Al and ^29^Si MAS NMR data are provided in the Supporting Information. Based on 25 catalyst preparations and measurements starting from the parent NH_4_Y, the standard deviation for [H_TOT_^+^] = 0.12.

**Table 1:** Summary of Characterization Data for HY Zeolites Used in This Study

catalyst	[H^+^_SD_] (mmol/g)	[H^+^_SC_] (mmol/g)	[H_TOT_^+^] (mmol/g)	[Na^+^]^a^ (mmol/g)	[H_TOT_^+^] + [Na^+^]	Si/Al^a^	Si/Al^b^	surface area^c^ (m^2^/g cat)
**HY**	1.6	1.4	3.0	1.2	4.2	2.6	2.8	880
**HY-3x**	2.1	1.5	3.6	0.5	4.1		2.7	
**HY-5x**	2.5	1.6	4.1	0.1	4.2	2.6	2.6	743
**HY-Na-1x**	0.8	1.4	2.2	2.0^d^	4.2			

a Measured via elemental analysis in ammonium form at Galbraith Laboratories.

b Measured via ^29^Si MAS NMR in ammonium form.

c Measured via nitrogen adsorption in ammonium form after exchange, drying, and mortar-pestle crushing.

d Measured from difference calculations on quantitative ^1^H NMR spectra.

The spectra in Figure 1 indicate that the HY, HY-3x, and HY-5x have essentially the same [H^+^_SC_] = 1.4–1.6 mmol/g but the [H^+^_SD_] increases by ca. 50% over the series. For the HY-5x, the total BAS density = 4.1 mmol/g, very near the 4.3 mmol/g expected for a Si/Al = 2.6–2.7. Indeed, when the measured NMR value of 4.1 mmol/g for all BASs in HY-5x is added to its residual Na^+^ = 0.13 mmol/g from Table 1, it is clear that all Al exists as framework Al, and that essentially all framework Al sites are converted to BASs in HY-5x. For HY and HY-3x, the BAS and residual Na^+^ ion sum to the very near the expected 4.3 mmol/g. Most importantly, the BAS density increase across the catalyst series results exclusively from an increase in [H^+^_SD_], resulting in HY-5x which has essentially the maximum BAS density expected for an HY catalyst with Si/Al = 2.6 and intact sodalite cages. Zeolite Y with Si/Al = 2.6 is the most common as-synthesized composition, and is the starting point for the preparation of many industrial catalysts. To our knowledge, this is the first report of a catalyst that meets these criteria, and which can serve to interrogate our fundamental understanding of acid site contributions in zeolites with multiple types of BASs in the absence of complicating structural features like defects, Lewis acid sites, and mesoporosity.

### Consequences for Hydrocarbon Reactivity

Two types of reactions that span a wide range of experimental conditions were chosen to probe the impact of sodalite cage acid sites on hydrocarbon reactivity. First, in situ ^2^H/^1^H exchange NMR experiments involving toluene-d_8_ and the HY, HY-3x, and HY-5x catalysts were used to determine the isotopic exchange rate constants at room temperature. This experimental approach has the advantage that signals from the catalyst acid sites and the reagent can be detected simultaneously, as well as detect any signals from defects that might form during reaction (Figure S6). Second, isooctane (2,2,4-trimethylpentane) cracking experiments were conducted at 400 °C with the same catalysts. Data from each type of experiment are in general agreement, as discussed below, even though one involves a low-energy barrier isotope exchange while the other involves hydrocarbon skeletal rearrangements. Again, toluene and isooctane are bulky reagents relative to the sodalite cage windows.

Figure 2 shows kinetic rate plots for the in situ H/D exchange experiment between the three HY catalysts and tolene-d_8_. As is known from the literature, room temperature H/D exchange between a zeolite and toluene only occurs at the toluene ring site, and not at the methyl position. While data was collected out to 240 min, as shown in Figure S7, Figure 2 shows short time data for clarity and fitting. Figure 2a shows a simple semilog plot for the growth of the toluene signal. Figure 2b shows a fit to the full exponential expression for the toluene signal intensity as a function of reaction time with an initial rate analysis assuming a pseudo-1st order reaction in [BAS], given the initial excess of toluene-d_8_ exchange sites. The fitting equation is provided in the Figure 2b caption and incorporates a time shift factor since the reaction begins before the first data point can be acquired. For this reason the linear extrapolations in Figure 2a do not intersect the *y*-axis at 0, but this does not limit accurate comparison of the respective rate constants. Similar rate constants were obtained with both fitting methods, and show significant increases up to a factor of 5, from the parent HY to the HY-3x and HY-5x catalysts. Again, it is important to clarify that [H^+^_SC_] is essentially the same in all three catalysts while only the [H^+^_SD_] increases. Such a result is not expected based on toluene’s inability to access the sodalite acid site.

**Figure 2:**
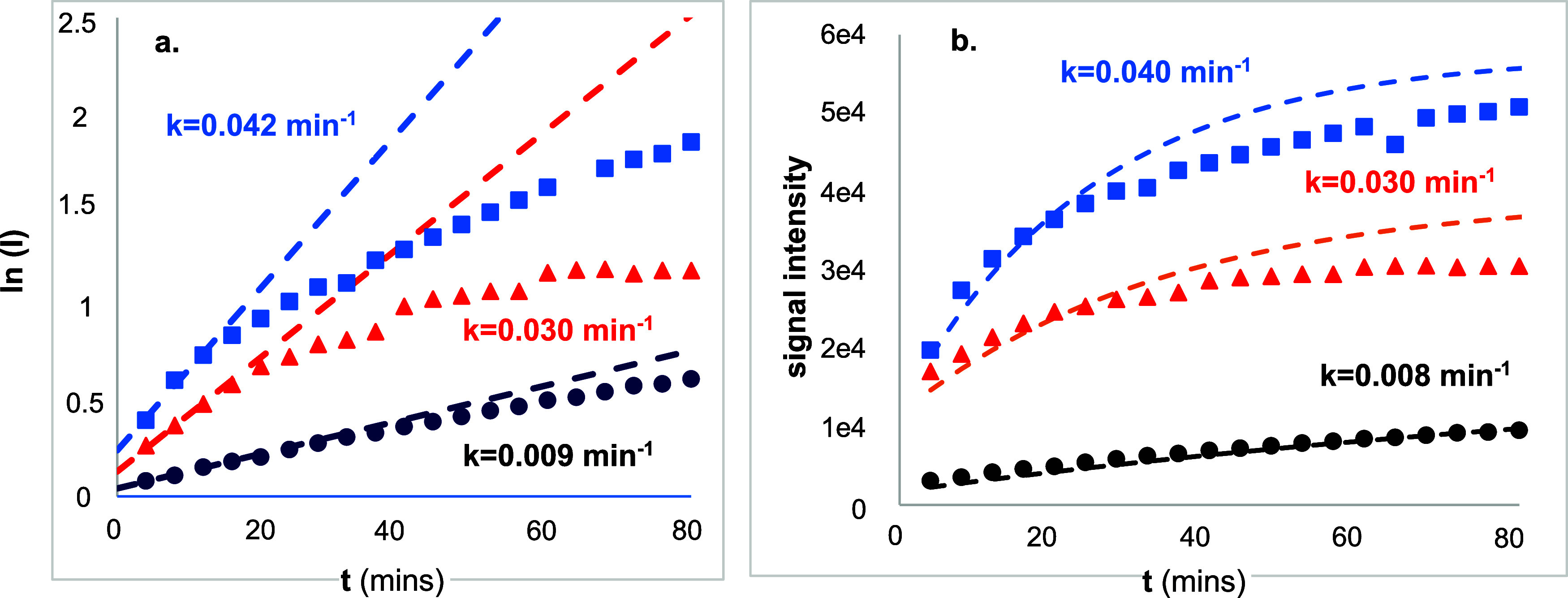
Raw data and kinetic analyses from in situ room-temperature ^1^H MAS NMR ^2^H/^1^H exchange between toluene-d_8_ and zeolites HY (•), HY-3x (▲), and HY-5x (■). (a) Ln­(I) vs time plot and linear regression analysis (dashed line) of the growth of the toluene aromatic ^1^H signal, where *I* = signal intensity in arbitrary units. (b) Fits (dashed line) to the raw intensity (points) vs time according to the equation *I*(*t*) = *I*(∞)­(1 – e^(−*k*(*t*+*c*))^), where the time constant *c* was used to correct for the onset of reaction prior to collection of the first data point. Typically, 8–10 min elapsed between reagent adsorption and the onset of data collection, and the fitting constant *c* were 12, 12, and 6 min for the HY, HY-3x, and HY-5x data in 2b, respectively.

Figure 3 shows conversion and reaction rate data for isooctane (i-C_8_) cracking at 400 °C for the same catalysts discussed in Figure 2. In agreement with the room-temperature isotopic exchange data discussed above, the conversion for isooctane cracking increases by 6-fold for HY-5x vs the parent HY, with HY-3x exhibiting intermediate performance. Moreover, the rate per gram of HY-5x catalyst increases 10 times relative to the parent HY, with HY-3x again exhibiting intermediate behavior. These striking results indicate a dominant role for [H^+^_SD_] with respect to catalyst performance, since the [H^+^_SC_] remains constant across the catalyst series. The sodalite cages were clearly intact in isotopic exchange experiments of Figure 2, as explained above and supported by the in situ spectra. For the high-temperature cracking experiments summarized in Figure 3, the catalysts were never exposed to moisture during the catalyst loading or reaction, as catalyst preparation in the reactor was carried out under 25 Torr vacuum, ensuring that sodalite units and their associated acid sites were intact. Selectivity does not change across the three catalysts, as shown in Figure S8 by the essentially constant C_4_/C_4_^=^ ratios. At the low conversions (8%) and low partial pressures for the data shown in Figure S8, the product distribution was primarily composed of methane, propene, butane, and butene. In comparing the rate and conversion results in Figures 2 and 3, consistent trends are clearly observed in both types of experiments. Indeed, a pseudo first-order rate-law analysis using both the high-temperature isooctane cracking rates under isoconversion conditions in conjunction with the [H^+^_SD_] and [H^+^_SC_] values enumerated in Figure 1 yields a reaction order of 2 with respect to [H^+^_SD_]. The rate order fits are shown in Figure S9, acquired under the same conditions as those in Figure S8. In all experiments [H^+^_SC_] was constant, and reagent was assumed to be in excess. In total, the data from two different reactions involving bulky hydrocarbons and extremely different reaction conditions in HY zeolites with the same number of supercage BASs indicate that the varying amounts of BASs in sodalite cages ([H^+^_SD_]) are the dominant contributor to conversion and reaction kinetics.

**Figure 3:**
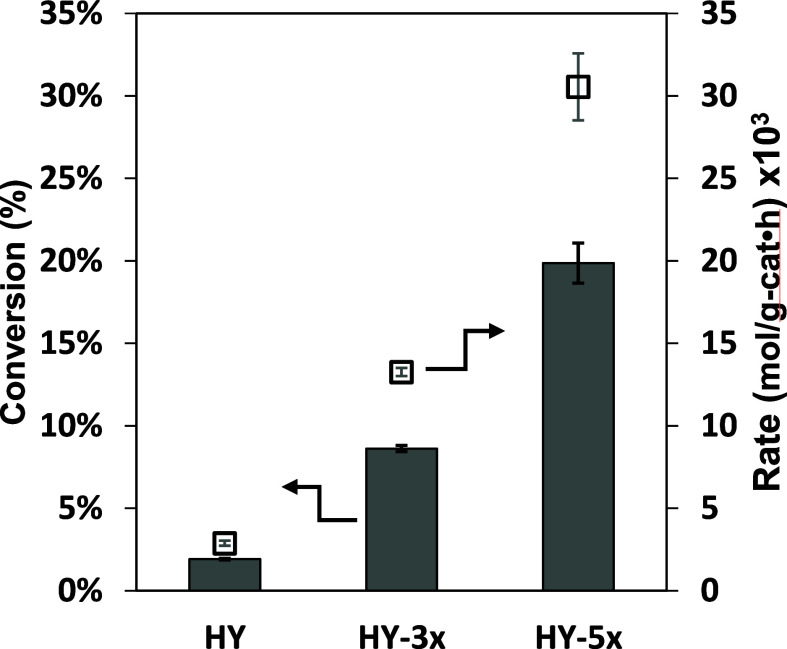
Conversion (bars/left axis) and reaction rates (squares/right axis) per gram of catalyst for isooctane (i-C_8_) cracking over the indicated catalysts at 400 °C in a flow reactor. Error bars reflect deviation in activity over 225 min time on stream. Catalysts were prepared under vacuum in the reactors at 25 Torr, with i-C_8_ introduced at 0.5 mL/h into 80 SCCM flowing helium.

### Sodalite-Specific Modifications Tune Catalyst Reactivity

Using the insights from the controlled-variation [H^+^_SD_] experiments described above, additional modifications of the types of cations or other species that reside within or interact with the sodalite cages were conducted. Based on these findings and previous literature,^45–50^ it is expected that small amounts of industrially relevant exchangeable cations like rare-earths could be selectively introduced into the sodalite cage. Further, as discussed in the previous section, the recognition that small amounts of water prefer to hydrolyze sodalite cage bonds can also be used to probe the impact of those sites on overall catalyst performance. Figure 4 demonstrates the wide range in TOF accessible for the same starting HY catalyst, based solely on varying the type and number of cations in the sodalite unit, while holding the supercage BAS density constant.

**Figure 4:**
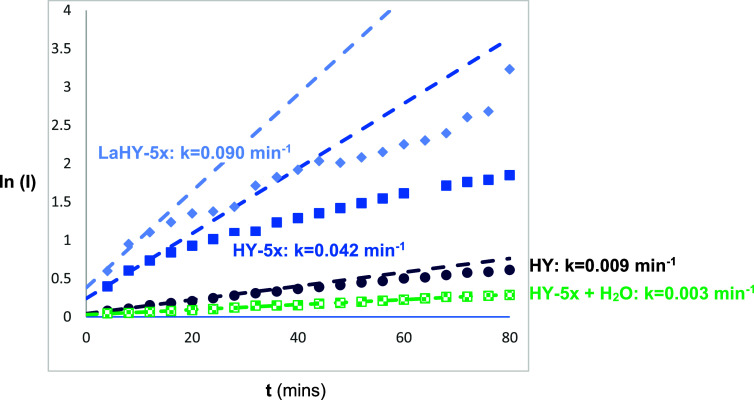
Raw data (points) and initial-rate kinetic analyses (dashed line) from in situ ^1^H MAS NMR ^2^H/^1^H exchange at room temperature between toluene-d_8_ and zeolites ordered from bottom of figure to top as HY-5x + 0.6 equiv coadsorbed H_2_O (⊡); HY (•); HY-5x (■); La-HY-5x with La:Al_F_ = 0.05 (◆). As shown in Figure S10, the [H^+^_SD_] is reduced to 1.0 mmol/g for the La-HY-5x, as compared to 2.5 mmol/g for HY-5x.

Figure 4 compares results from the toluene exchange experiment for the parent material HY to HY-5x, as above, and also includes results for HY-5x to which either La or H_2_O has been introduced. First, the top two sets of data in Figure 4 show that the impact of selectively incorporating La^3+^ into the sodalite cages of HY-5x is to increase the reaction rate by more than a factor of 2. Recall from the discussion above that the HY-5x was the most active catalyst, with complete conversion of all framework Al atoms (Al_F_) into BASs and concomitant maximum [H^+^_SD_]. The spectra in Figure S10 demonstrate that La can be selectively incorporated into the sodalite units of HY-5x in relatively low amounts (0.05 La:Al_F_) while preserving the cage structure, as indicated by the lack of any silanol or aluminol signals in the 1.5–3 ppm region as discussed above. The data in Figure S10 are consistent with previously published reports detailing selective introduction of La into sodalite cages of HY, albeit on different HY catalysts than the HY-5x here.^29^ Most importantly, as detailed in Figure S10, *La incorporation in HY-5x reduces [H* ^*+*^_*SD*_ *] from 2.5 to 1.0 mmol/g without impacting [H* ^*+*^_*SC*_ *], and yet* Figure 4 *shows that the reaction rate for toluene isotope exchange increases by a factor of 2*. If the mechanism for enhanced reactivity in HY vs HY-3x vs HY-5x arises from increased proton density in sodalite units and concomitant proton shuttling, then replacing more than half of those H^+^_SD_ sites with La^3+^ would be expected to reduce reaction rates, not increase them as observed here. Figure S11 shows that increasing the [Na^+^] in sodalite cage sites according to the data in Table 1 leads to significant reactivity decreases as one examines the data for the series HY-5x to HY-Na-1x, consistent with an interruption of a proton-hopping network. Similarly, it is known that trace water increases proton shuttling or proton hopping.^51^ However, Figure 4 also shows that the controlled addition of trace water at a level of 1 water molecule for every two toluene molecules leads to over an order of magnitude *decrease* in reaction rates relative to the HY-5x without coadsorbed water, further suggesting that the origin of the marked increase in reactivity with increasing H^+^_SD_ in the HY-5x vs HY-3x vs HY does not arise from a simple proton shuttling mechanism as would be enhanced by the presence of water. The data in Figure 4 does clearly show that the addition of La^3+^ to an HY catalyst with maximum [H^+^_SD_] and overall maximum total [BAS] results in a large synergistic impact on reaction rates.

The conclusions from Figure 4 are supported by high-temperature isooctane cracking experiments on the same 0.05 La-HY-5x and HY-5x catalysts. As shown in Figure 5 below, selective introduction of La^3+^ into integral sodalite cages of HY-5x, and concomitant decrease in [H^+^_SD_], leads to significant conversion and reaction rate increases above that measured for the maximum proton density HY-5x catalyst. Indeed, the rate for La-HY-5x is more than twice that measured for HY-5x and more than an order of magnitude larger than for the parent HY, which is almost identical to the rate increases measured for the isotopic exchange experiments in Figure 4. The large increases in isooctane reactivity following selective incorporation of La into sodalite cages at the expense of the sodalite BASs suggests that the increased reactivity in HY-5x arises from strong synergies between the rare earth cation and proton sites, as well as potential for enhanced electric field gradients. Again, La incorporation in HY-5x reduces [H^+^_SD_] from 2.5 to 1.0 mmol/g but significantly increases conversion and reaction rate relative to HY-5x in both reactions.

**Figure 5:**
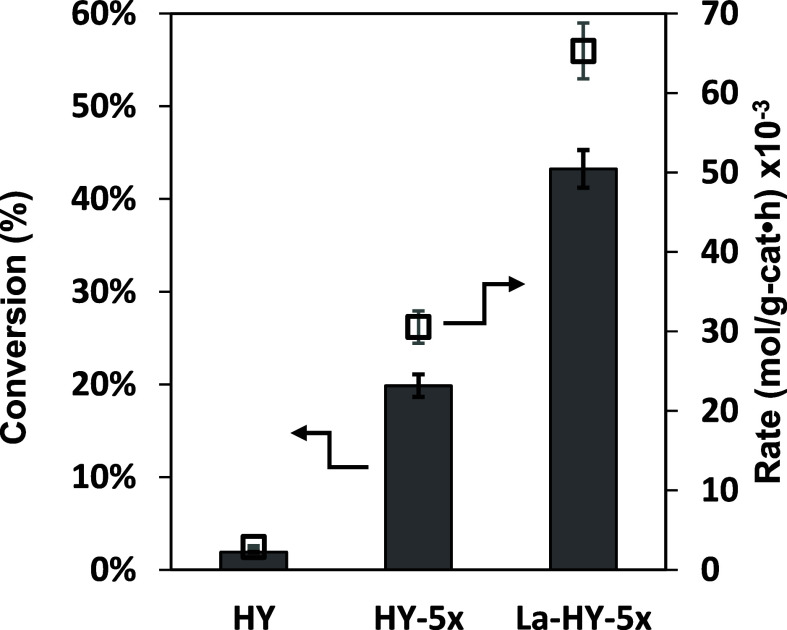
Conversion (bars/left axis) and reaction rates (squares/right axis) per gram of catalyst for isooctane (i-C_8_) cracking over the indicated HY and La-HY catalysts at 400 °C in a flow reactor. Error bars reflect deviation in activity over 225 min time on stream. Catalysts were prepared under vacuum in the reactors at 25 Torr, with i-C_8_ introduced at 0.5 mL/h into 80 SCCM flowing helium.

### Proposed Mechanism of Sodalite Proton Action

The experimental results above suggest that a simple proton-hopping mechanism may not be the only mechanism to explain the increased catalyst reactivity with increasing [H^+^_SD_] in the HY-3x and HY-5x catalysts compared to the parent HY. It was previously shown that sodalite acid sites react with benzene in USY catalysts via a proton-shuttling mechanism, although the integrity of the sodalite cages was compromised due to framework hydrolysis.^26^ Also, it is known from ^17^O labeling work that oxygen bridges are labile in the presence of liquid water, which can provide a dynamic pathway for sodalite cage opening and closing in the presence of water thereby allowing access to H^+^_SD_ sites.^52,53^ However, such conditions are not applicable in any of the gas-phase experiments on dehydrated zeolite catalysts employed here, as shown by the lack of any defect signals in the quantitative NMR and the consistent XRD and SEM data in Figures S5 and S12. Further, the coadsorption of water with toluene in the kinetic trials lead to dramatically lower reaction rate constants. Finally, it has been clearly demonstrated in the literature by using ^129^Xe NMR experiments that the reagents used in this study cannot enter the sodalite cages at the room-temperature conditions used for the toluene reactions in Figures 2 and 4.^6,55^ Under high-temperature conditions like those used for the isooctane cracking, there is precedence in the literature for thermally induced expansion of some cage and channel openings, as recently reviewed, albeit not specifically for sodalite cage openings.^54^ In situ FTIR was used to probe sodalite cage integrity following adsorption of the bulky base 2,6-di*tert*-butylpyridine (DTBPy). Figure S13 shows FTIR spectra for HY and HY-5x following high-temperature dehydration, and in agreement with the ^1^H MAS NMR results, no signals from any silanol group defects are observed in the 3700 cm^–1^ region. Figure S14 shows, in agreement with prior work,^56^ that this bulky amine does access supercage sites in HY due to the complete attenuation of the [H^+^_SC_] signal at 3640 cm^–1^. However, the majority of the [H^+^_SD_] signal remains, consistent with the inability of the bulky probe to form the necessary protonated adduct due to size exclusion, even after holding the temperature at 450 °C for 1 h. In total, all of the data herein along with cited literature data discussed above indicates that the sodalite cage remains intact under the experimental conditions used in this study.

In order to clarify the mechanism by which sodalite acid sites are able to unexpectedly impact overall catalyst performance with such significance, DFT (density functional theory) calculations were completed on HY in the absence and presence of a benzene reagent. Benzene was chosen over toluene, as it easier to model, and the experimental data only showed evidence for H/D exchange at aromatic ring positions and not at the methyl group. The results imply that the sodalite sites exhibit some degree of framework flexibility such as rotation of the sodalite OH into the supercage or through a proton migration mechanism from a sodalite facing oxygen to a supercage facing oxygen. The proposed mechanism for framework flexibility is shown in Figure 6A. In one scenario, [H^+^_SD_] can migrate past the framework aluminum to a connected oxygen and reach the supercage forming [H^+^_SC_]; however, this unassisted proton transfer has a barrier of 85 kJ/mol. In an alternate scenario, [H^+^_SD_] can rotate through a small window inside the hexagonal bridge and rest on the other side forming [H^+^_SC_], accessible to reactants in the supercage. This proton rotation results in a less stable and more acidic proton with higher chemical potential. The activation barrier required for this [H^+^_SD_] rotation into a supercage accessible position is about 51 kJ/mol. This barrier can be reduced by a nearby benzene molecule in the supercage, to 45 kJ/mol as shown in Figure 6B. Note water molecules in the sodalite cage can form hydrogen bonds with the [H^+^_SD_], increasing the latter’s stability and leading to an energetic penalty of 75 kJ/mol for proton rotation; this increased energy cost reduces the population of these accessible and highly active sites and provides an explanation for the lower reaction constants when water was introduced in the experiments (Figure 4).

**Figure 6:**
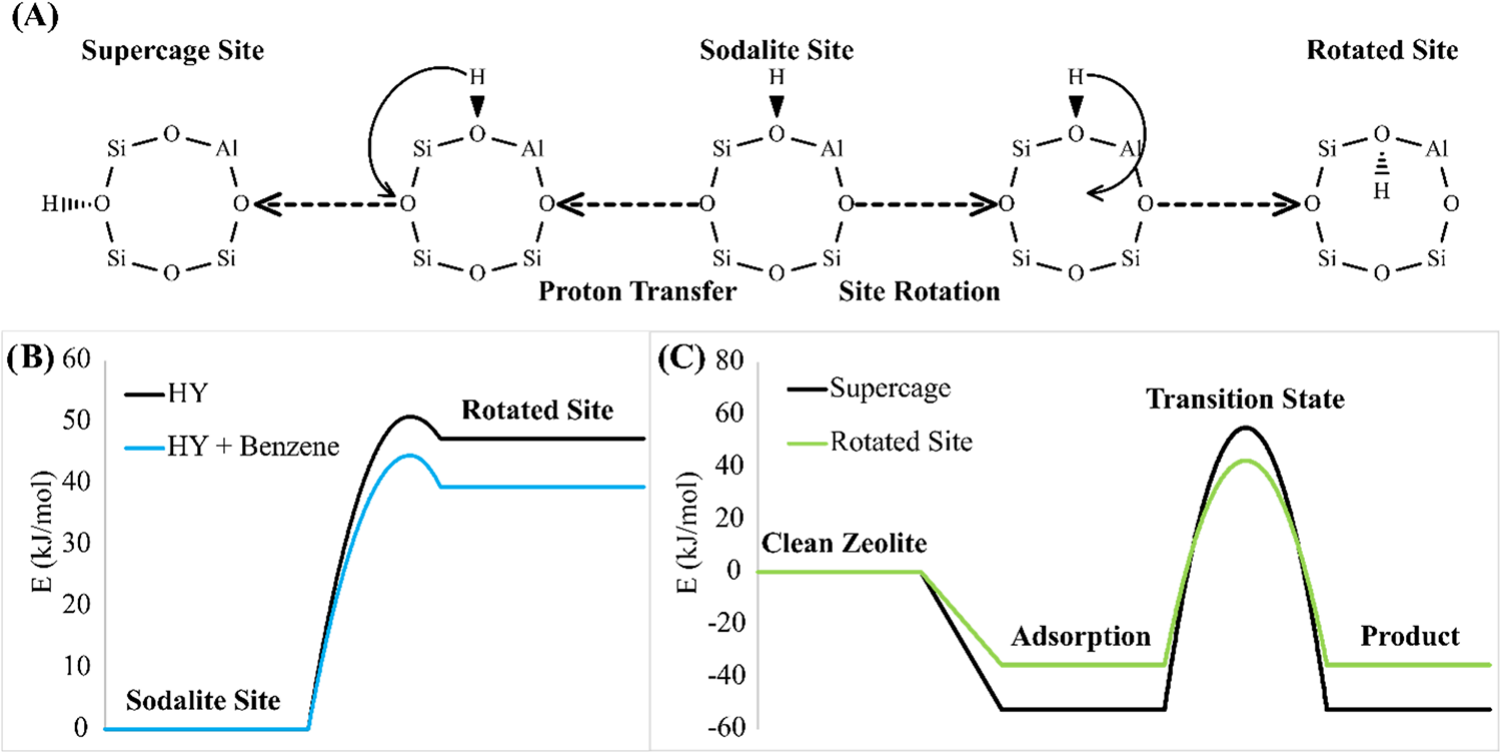
DFT calculations of proton rotation and its H/D exchange activity. (A) Schematic showcasing how sodalite sites can become an accessible site in supercage; the left side is for proton migration into the supercage, while the right side shows the pathway for site rotation. (B) Energetics for the site rotation mechanism both in the presence and absence of a proximal benzene in the supercage. (C) Reaction profile for the benzene H/D exchange starting from gas-phase benzene and a supercage site or a rotated sodalite site. Benzene adsorbs on the active site, followed by the H/D exchange transition state, resulting in the H/D exchanged benzene product.

Next, the activation barrier for H/D exchange was calculated. Figure 6C shows the reaction pathway for benzene H/D exchange over both a conventional [H^+^_SC_] and a rotated site. First, benzene adsorbs at the rotated site, and despite higher acidity, this rotated site binds benzene more weakly due to its position at the cage window making it difficult to access due to steric hindrance. Next, the BAS proton transfers to the benzene, exchanging with deuterium. Here, both the intrinsic and apparent activation barriers for the rotated site are lower than H/D exchange on a traditional [H^+^_SC_]; the intrinsic activation energies is 78 kJ/mol over the rotated site as opposed to 108 kJ/mol over the traditional [H^+^_SC_]. The latter value is comparable to a prior DFT study (∼100 kJ/mol)^2^. At the transition state, the oxygen hosting the rotated site retreats into the sodalite cage, which causes the deuterium to preferentially return to a neighboring oxygen inside the supercage. To complete the catalytic cycle, this site or another nearby BAS should transfer its proton across the boundary into the sodalite cage, recovering [H^+^_SD_]. It is anticipated that if the sodalite cage is crowded by other cations, e.g., La^3+^ (Figures 4 and 5), such recovery can be suppressed resulting in a longer lifetime of the rotated proton site in the supercage. Overall, this proposed catalytic cycle provides a mechanism that would lead to increased activity when increasing sodalite BAS density, and is not inconsistent with recent proposals in the literature about zeolite framework flexibility.^54^

## Conclusions

In the absence of postsynthetic treatments to “open” up sodalite units in zeolite HY, the relative reactivity of acid sites in those sterically occluded locations has been dismissed in the literature. Further, preparation of high-framework Al content zeolite Y in which essentially all of the as-synthesized framework Al translates into Brønsted sites has not been documented in the literature, with most reported measures of total [BAS] well below the expected ca. 4 mmol H^+^/g catalyst. Here, a combination of catalyst preparation methods yielding catalysts for the first time with [BAS] up to 4.1 mmol H^+^/g catalyst, with quantitative NMR spectroscopy, flow-reactor, and computational experiments has demonstrated that BASs in intact, undamaged sodalite cages can define and control catalyst reactivity in HY catalysts. The H^+^_SD_ sites are shown to be highly reactive, and for catalysts with fixed amounts of H^+^_SC_, reaction rates depend significantly on the concentration of acid sites in the intact sodalite units with an apparent reaction order equal to 2. These results were consistent in two very different reaction types involving bulky hydrocarbons that are too large to enter the sodalite cage, i.e., high-temperature isooctane cracking and room-temperature toluene isotopic exchange experiments. The impact of the cation identity in intact sodalite units was further confirmed by La^3+^ exchange experiments, in which only H^+^_SD_ protons were replaced by La, leading to very large increases in reaction rates above that observed for the catalysts with the theoretical maximum values of [BAS] = 4.1 mmol/g. DFT results support a mechanism by which Bronsted acid sites within zeolite Y exhibit flexibility in the form of site rotation across the supercage/sodalite walls, which is not inconsistent with very recent proposals regarding zeolite framework flexibility.^54^ Indeed, while the low-temperature H/D exchange experiments are conducted in a regime in which framework dynamics are not expected based on the literature,^6,55^ these cannot be completely excluded for the high-temperature cracking experiments. The rotated sites have increased activity and are partly responsible for the observed increases in reaction rates with increasing numbers of sodalite protons. The propensity for sodalite site “rotation” increases with increasing concentration of H^+^_SD_, as clearly shown by the reaction results with varying [H^+^_SD_], presumably due to the decreasing distance between positive charged centers in the sodalite units. In total, these results on one of the most widely used zeolite catalysts reveal a new way of interpreting the action of BASs as a function of their site geometries, and further demonstrate that the most commonly used methods for measuring acid site density may be undercounting, by a significant amount, the impact of “hidden” acid sites.

## Supplementary Material



## Notes

Additional information including XRD data, SEM data, MAS NMR data, in situ FTIR data, and isooctane reaction data (PDF)

## §

Chemical Sciences Division, Oak Ridge National Laboratories, Oak Ridge, Tennessee 37831, United States

## ∥

Department of Chemical and Biological Engineering, Tufts University, Medford, Massachusetts 02155, United States
