# Supplementary material for: Zeolite Catalysts Prepared with Maximum Brønsted Acidity Reveal a Dominant Contribution from Inaccessible Sites
Source: J Am Chem Soc. 2026 Jun 26;148(26):27975–84. doi: 10.1021/jacs.6c10814 (PMC13352632; doi:10.1021/jacs.6c10814)
Supplement: Supplementary file 1 [file ja6c10814_si_001.pdf]

Supporting Information

Zeolite Catalysts Prepared with Maximum Brønsted Acidity Reveal a Dominant Contribution from Inaccessible Sites

Omio Rani Das, Ismaeel Alalq, Jacob Crouch, Anya Zornes,♦ Steven Crossley, Bin Wang,# and Jeffery L. White*

***Corresponding Author**

Jeffery L. White, 420 Engineering North, School of Chemical Engineering, Oklahoma State University, Stillwater, Oklahoma 74078, United States; email: ***jeff.white@okstate.edu***

Authors

Omio Rani Das, 420 Engineering North, School of Chemical Engineering, Oklahoma State University, Stillwater, Oklahoma 74078, United States

Ismaeel Alalq, School of Sustainable Chemical, Materials, and Biological Engineering, University of Oklahoma, Norman, Oklahoma 73019, United States

Jacob Crouch, School of Sustainable Chemical, Materials, and Biological Engineering, University of Oklahoma, Norman, Oklahoma 73019, United States

Anya Zornes, 420 Engineering North, School of Chemical Engineering, Oklahoma State University, Stillwater, Oklahoma 74078, United States

Bin Wang, School of Sustainable Chemical, Materials, and Biological Engineering, University of Oklahoma, Norman, Oklahoma 73019, United States

Steven Crossley, School of Sustainable Chemical, Materials, and Biological Engineering, University of Oklahoma, Norman, Oklahoma 73019, United States

♦Present address: Oak Ridge National Laboratories, Oak Ridge, TN

#Present address: Department of Chemical and Biological Engineering, Tufts University, Boston MA

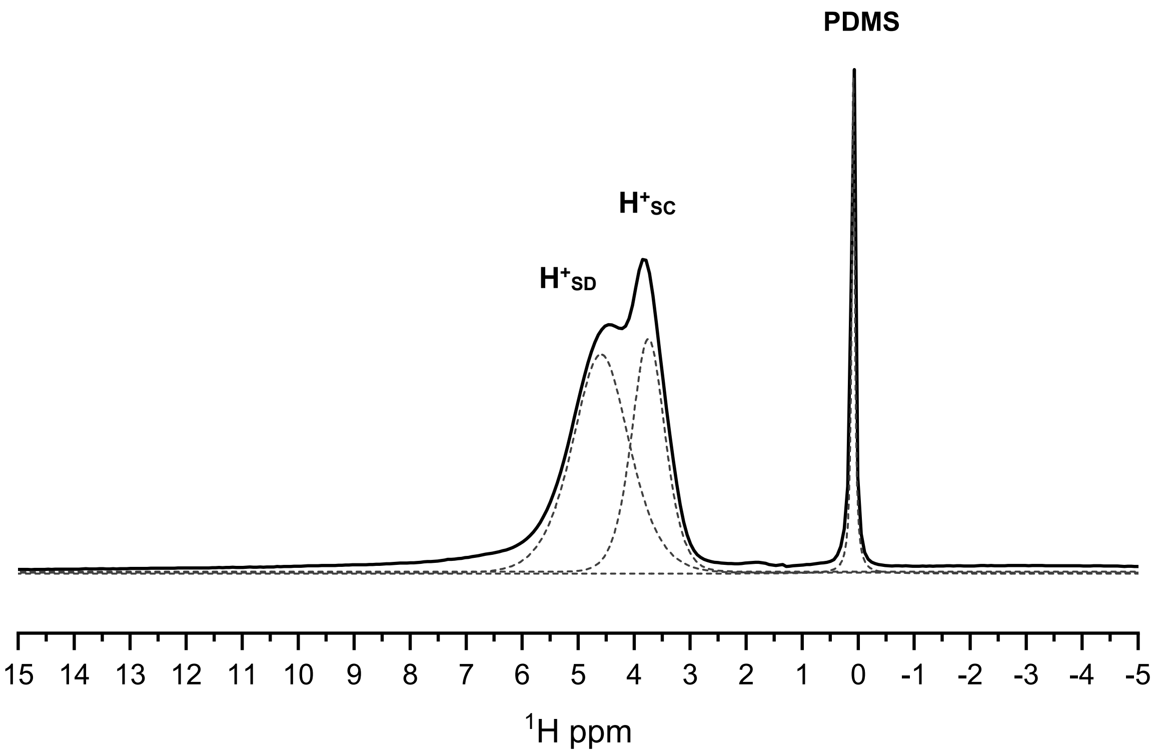

Figure S1. ^1H MAS NMR spectra of HY-5x, demonstrating the simple deconvolution and fitting to quantify the BASs in sodalite and supercage sites for all HY samples, as well as the peak from the internal PDMS standard.

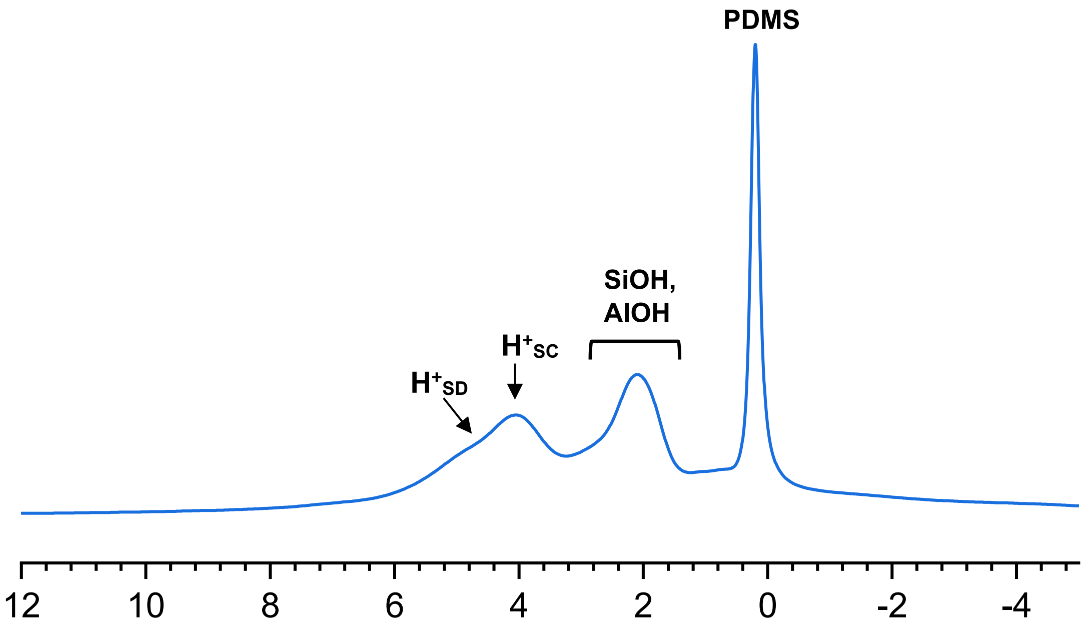

Figure S2. ^{1}H MAS NMR spectra of HY acquired after deionized water dissolution and followed by dehydration under vacuum demonstrating increased non-framework and framework defect SiOH and AlOH groups indicated by the signals in the 1.5-3 ppm region, with concomitant decrease in BASs in marked contrast to the catalysts shown in Figure 1. For this catalyst, the total (H^+_{SC} + H^+_{SD}) = 0.6 mmol/g.

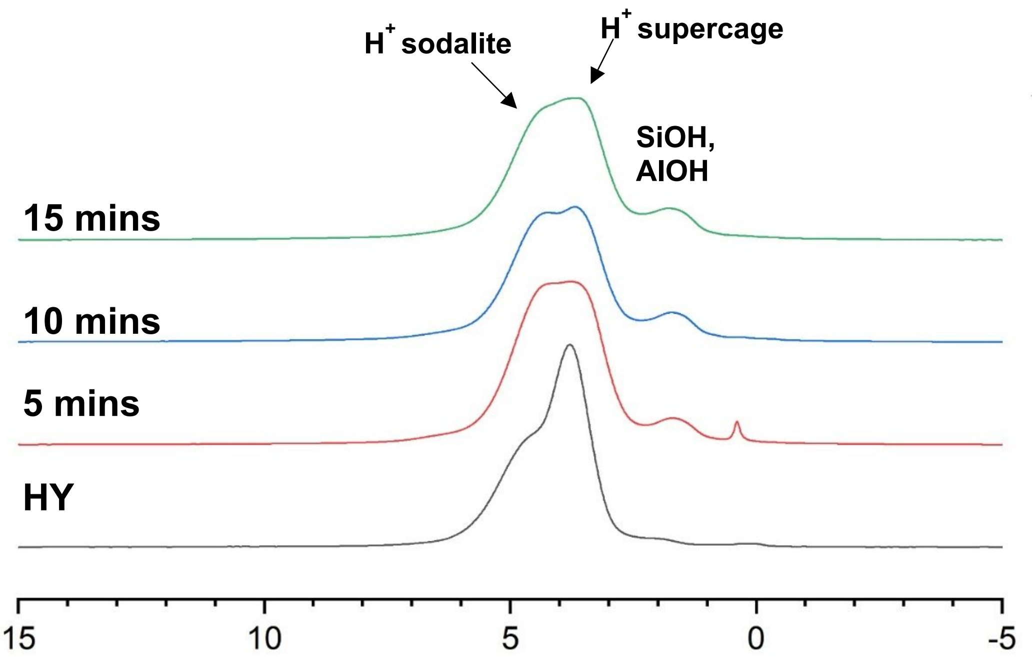

Figure S3. Representative ^{1}H MAS NMR spectra of HY following time-dependent NH_4F etching following the method of Reference 6, showing appearance of expected SiOH and AlOH signals due to framework hydrolysis.

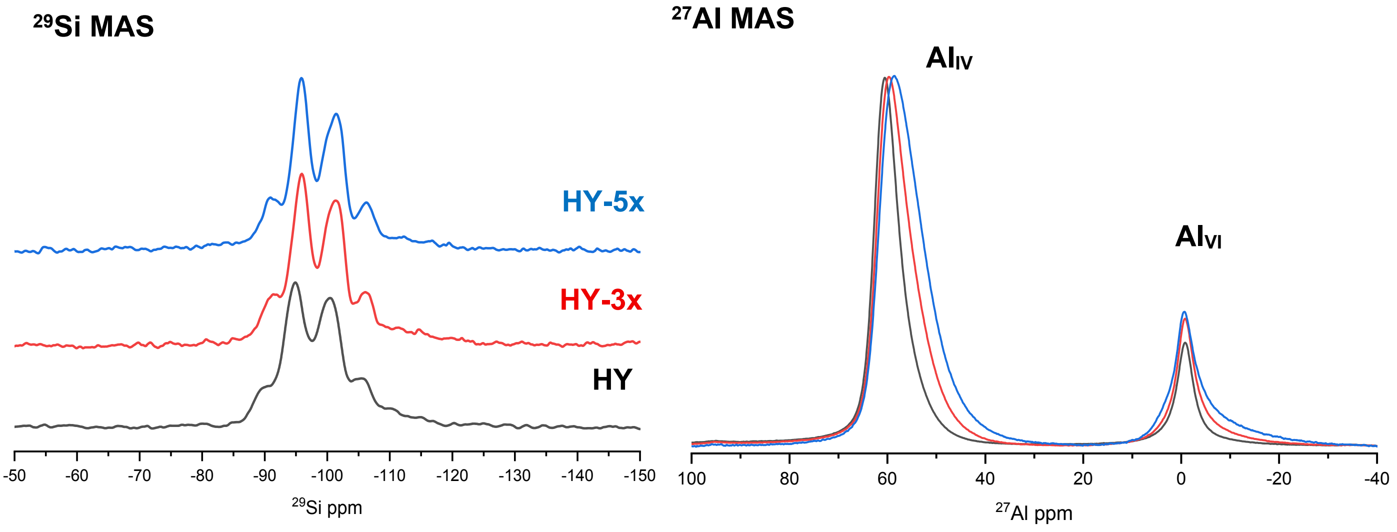

Figure S4. Representative ^{29}Si and ^{27}Al NMR spectra of HY (black trace), HY-3x (red trace), and HY-5x (blue trace). The calculated Si/Al from the HY-5x is 2.65. The 0-ppm peak in the ^{27}Al spectra arises from framework-bound octahedral Al atoms resulting from partial but reversible framework hydrolysis after catalyst exposure to ambient moisture that is required to acquire the ^{27}Al spectrum, and which has been discussed extensively by van Bokhoven and coworkers in References 12 and 14. Importantly, such species *are not* present during the in-situ toluene reactions or the isooctane cracking reactions using dry catalysts, as shown by the lack of signals in the 2-3 ppm region of the ^{1}H MAS NMR spectra.

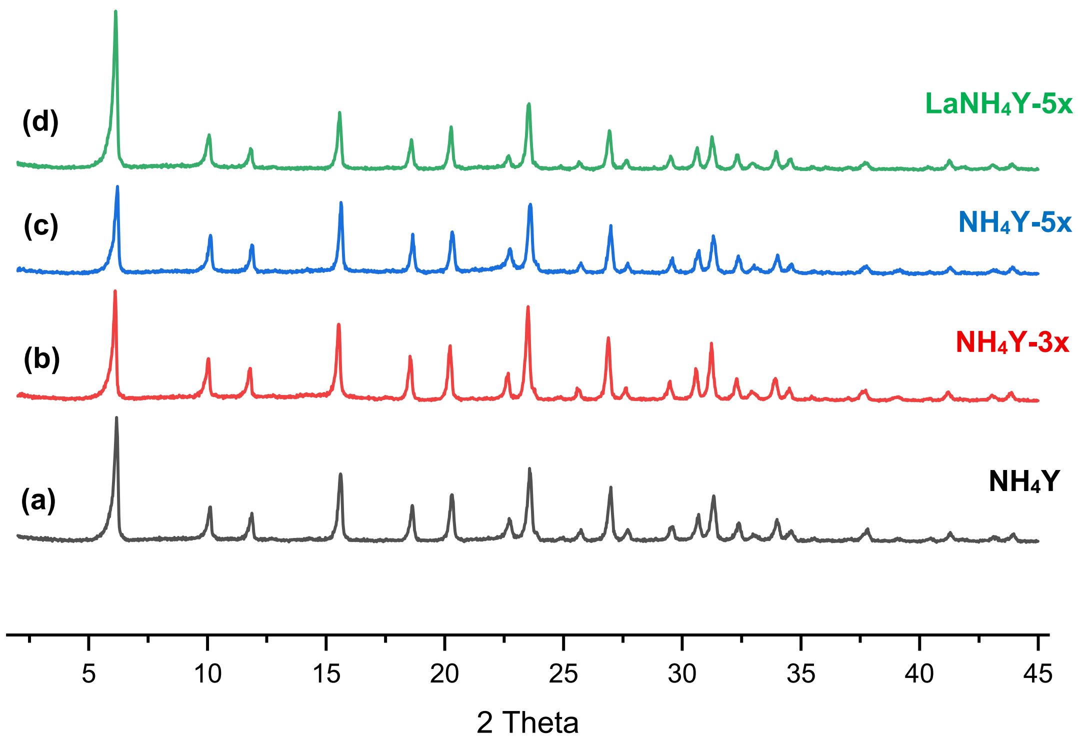

Figure S5. X-ray powder diffraction patterns of **(a)** parent NH_4Y with percent crystallinity (%xtal) = 99; **(b)** NH_4Y-3x: %xtal = 98 ;**(c)** NH_4Y-5x: %xtal = 98; **(d)** NH_4Y-5x exchanged with La^{3+}: %xtal = 99

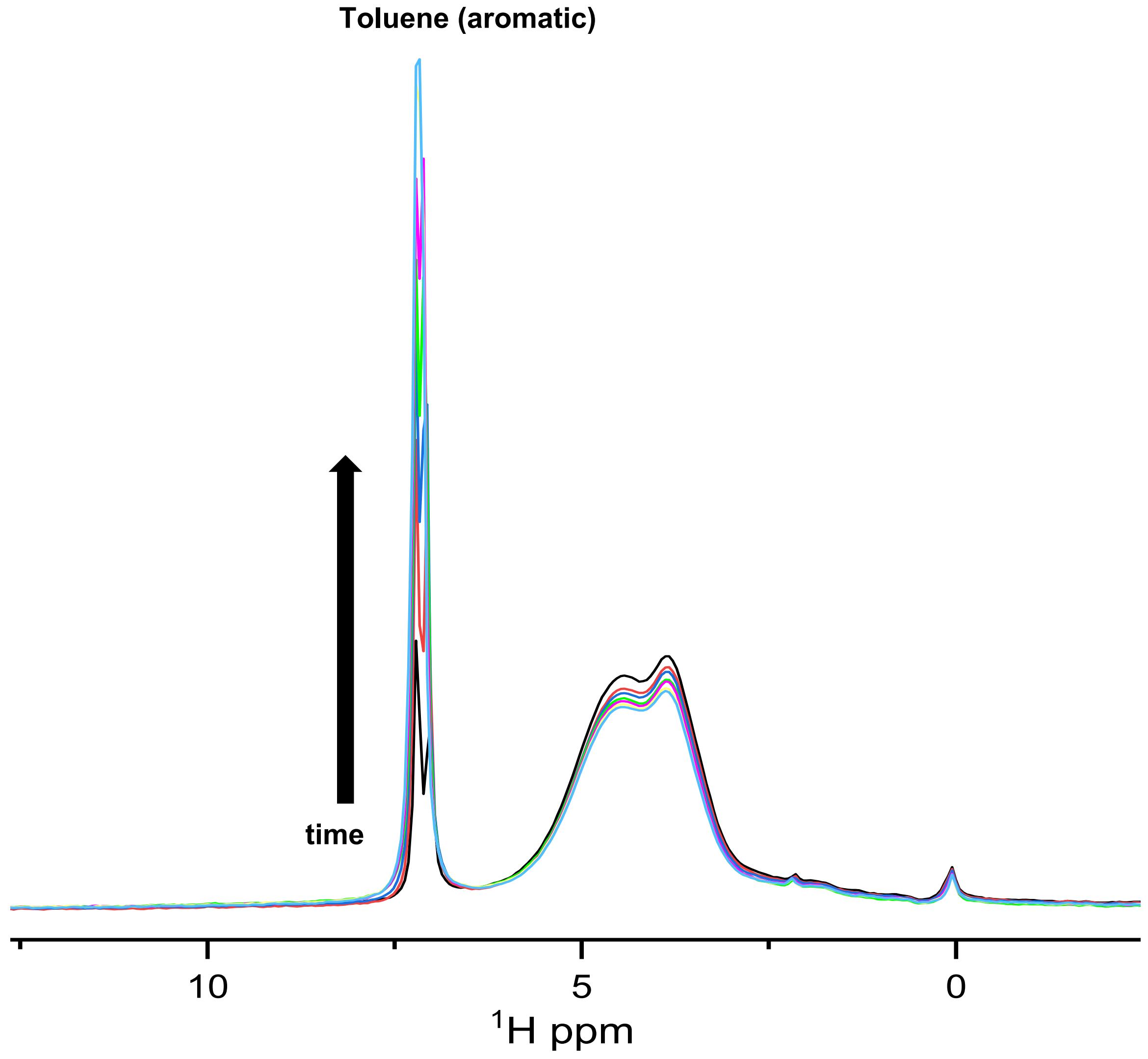

Figure S6. Selected spectra for H/D exchange of toluene-d_8 and HY-5x vs. time, demonstrating growth of toluene aromatic signal at 7 ppm whose integrated area was used in the rate plots, and the absence of exchange at the toluene CH_3 site.

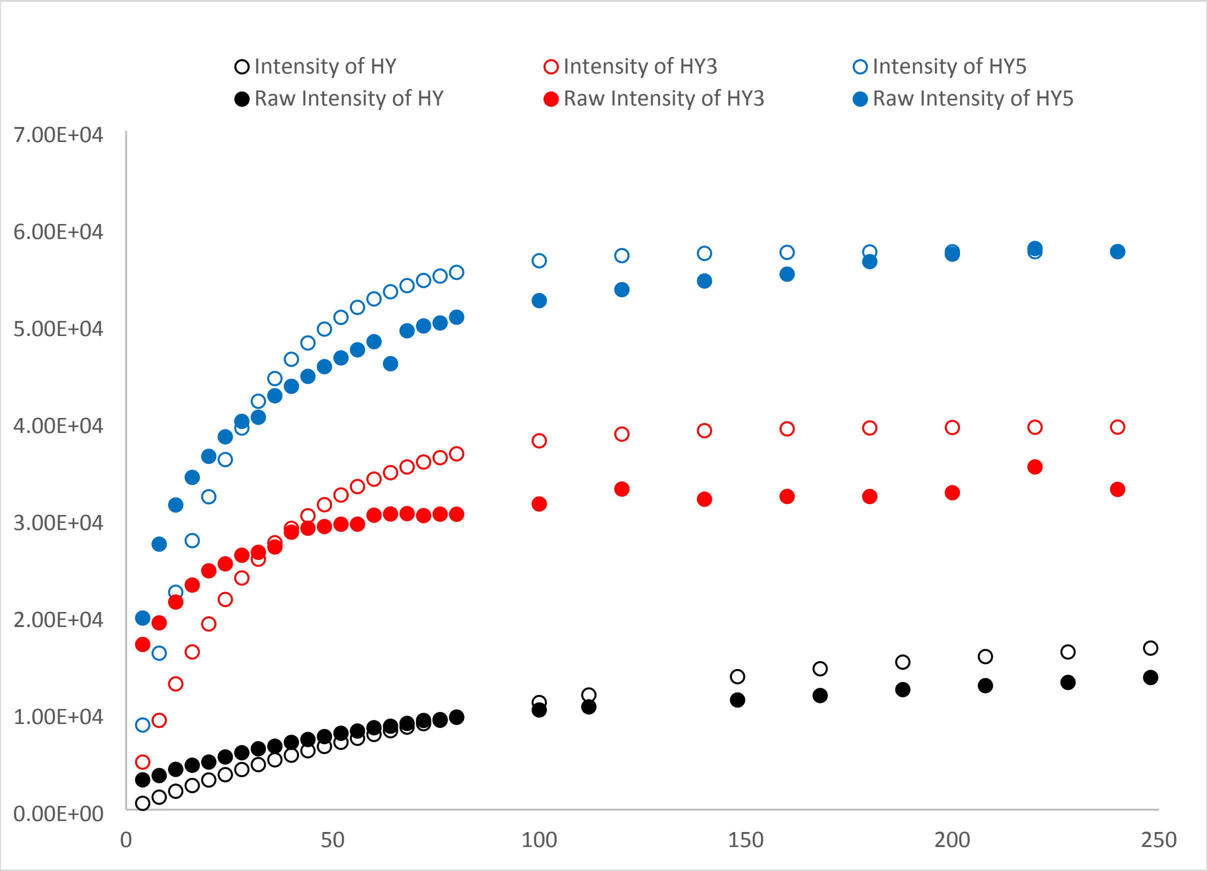

Figure S7. Complete time series for the raw intensity (filled points) of the toluene aromatic peak in H/D exchange reactions between toluene-d_8 and the HY catalysts as indicated. Fits to the data (open points) were obtained using the equation $I(t) = I(\infty)(1\text{-}e^{-(kt)})$. The raw data points do not intercept the y axis at 0 due to reaction onset prior to acquisition of the first data point, which in typical experiments results in a time offset of 5-10 mins. This can be accommodated using a time factor addition to the exponential fit as discussed in the main text.

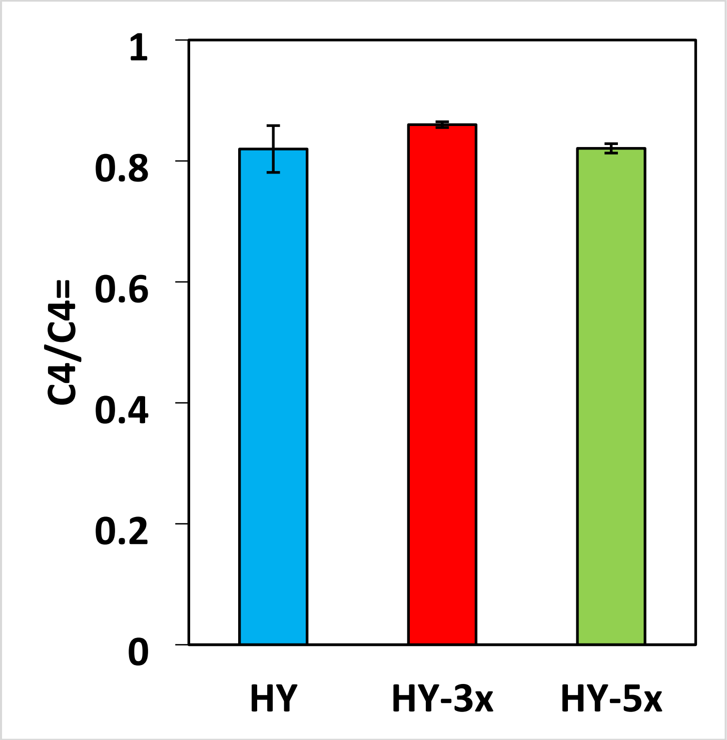

Figure S8. Summary of selectivity data for the three HY catalysts expressed as the $C_4/C_4^=$ in the product stream. At the low conversions (8%) and low partial pressures used here, the product distribution was primarily composed of methane, propene, butane, and butane. Catalyst mass was adjusted to achieve similar conversion for each of the three catalysts, ranging from 15-40 mg.

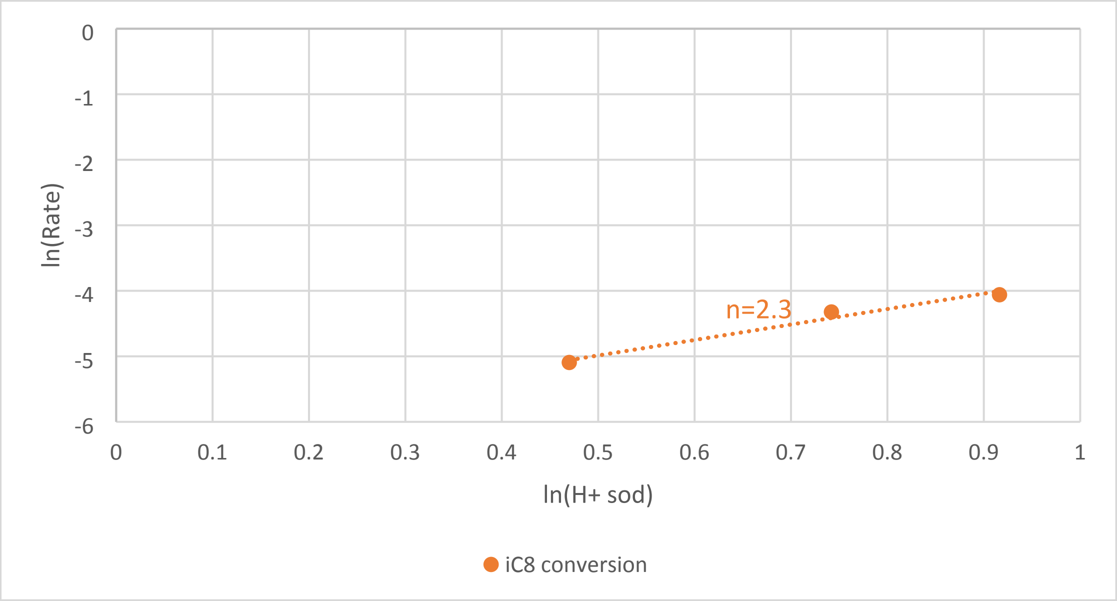

Figure S9. Approximate rate order analysis in [H_{SD}^{+}] for HY, HY-3x, and HY-5x catalysts used in isooctane cracking experiments with rate constants acquired at similar conversions (8%), showing an approximate 2nd-order dependence on [H_{SD}^{+}]. As discussed in the main text, [H_{SC}^{+}] is assumed to be a constant, and the isooctane concentration is assumed in excess relative to [H_{SD}^{+}].

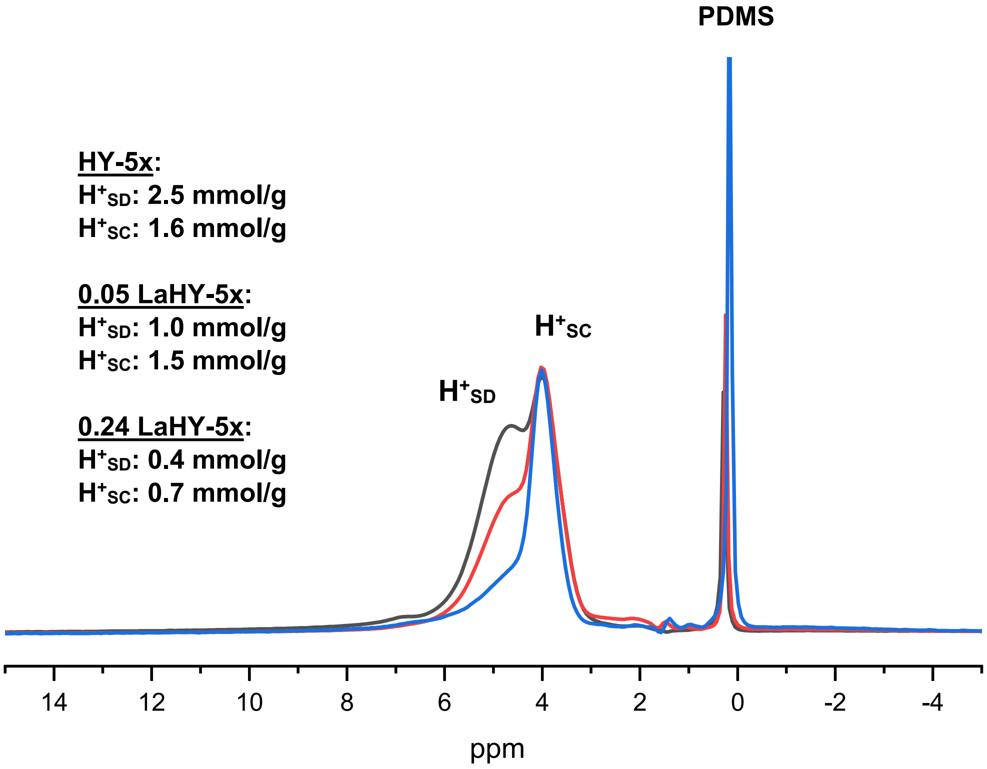

Figure S10. ^{1}H MAS NMR spectra of HY-5x (black), 0.05 La-HY-5x (red), and 0.24 La-HY-5x (blue), demonstrating that La can be selectively incorporated into sodalite units at low amounts (0.05 La: Al ratio La-HY-5x) while preserving sodalite cage structure as indicated by the absence of silanol or aluminol signals in the 1.5-3 ppm region. Spectra are normalized to the same height of the supercage acid site peak at 3.9 ppm.

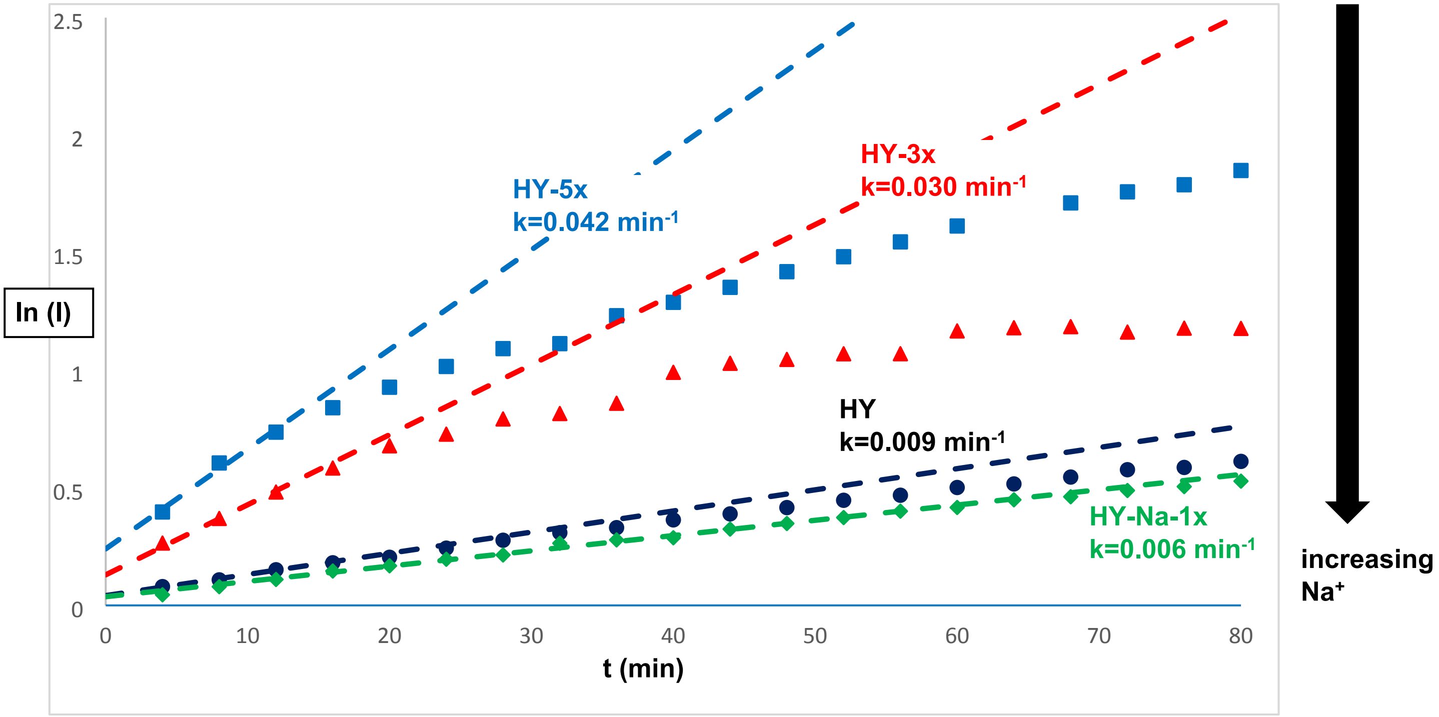

Figure S11. Toluene-d_8/HY exchange data (ln(I) vs. t) for the catalysts as labeled, i.e. the same catalyst series as shown in Figure 1 of the text, demonstrating the significant decrease in the turnover frequency relative to HY-5x with increasing [Na^+]. The [Na^+] in (mmol/g catalyst) are given in Table 1 of the main text.

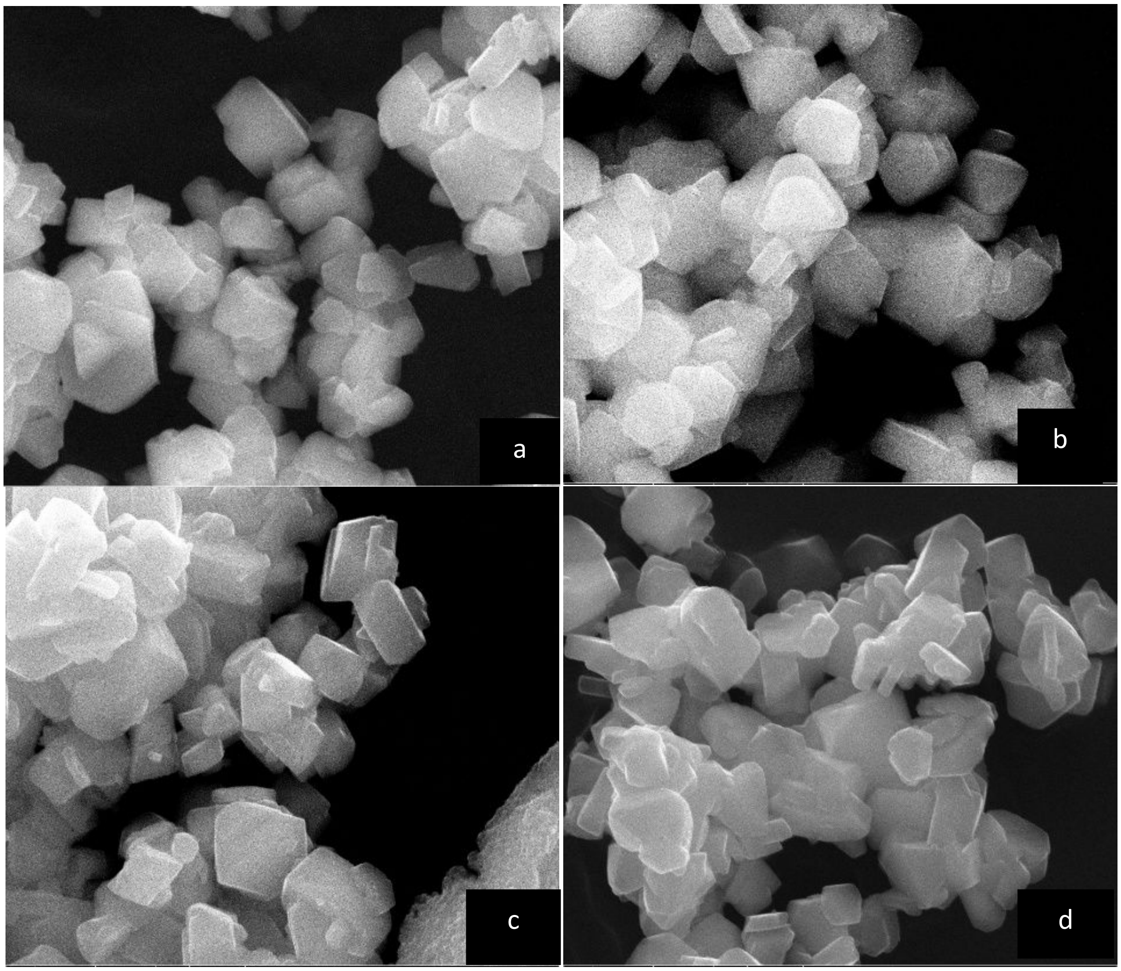

Figure S12. Scanning electron microscopy (SEM) images presented with a horizontal field width (HFW) of 5 μm for **(a)** parent NH_4Y; **(b)** HY ;**(c)** NH_4Y-5x; and **(d)** HY-5x.

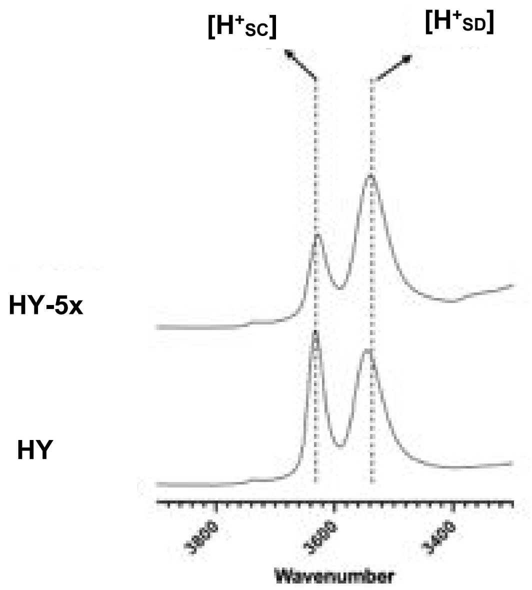

Figure S13. FTIR spectra of HY and HY-5x, as labeled, acquired without exposure to atmospheric moisture showing increased [H^+_{SD}] in the latter, and also showing characteristic absence of silanols defect signals near 3700-3800 cm^{-1} in agreement with NMR data discussed in the text. These spectra were acquired following heating to 450°C.

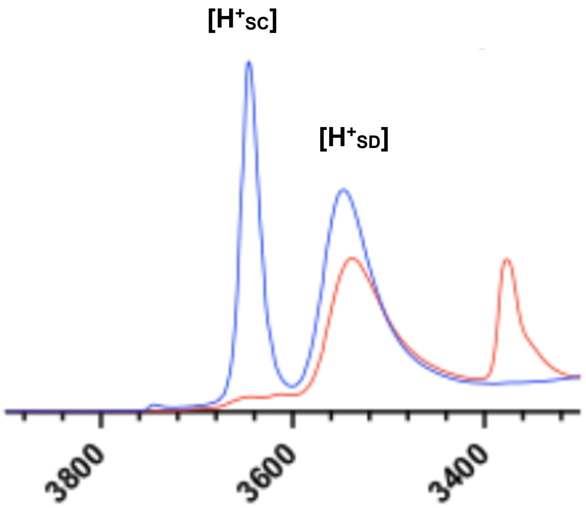

Figure S14. Overlay of in-situ FTIR spectra of HY before (blue trace) and after (red trace) 2,6-di-tert-butylpyridine (DTBPy) adsorption and exposure at 450°C for one hour. Note the complete attenuation of the signal associated with [H^+_{SC}] sites but only minimal attenuation of the [H^+_{SD}] signal, and the absence of any silanol defect signals near 3700cm^{-1} in agreement with NMR data discussed in the text and in Figure S13.
